# In-Hospital Complications of Coronary Artery Bypass Graft Surgery in Patients Older Than 70 Years

**DOI:** 10.15171/jcvtr.2015.13

**Published:** 2015

**Authors:** Naser Safaie, Hossein Montazerghaem, Ahmadreza Jodati, Nasrollah Maghamipour

**Affiliations:** ^1^ Cardiovascular Research Center, Tabriz University of Medical Sciences, Tabriz, Iran; ^2^ Cardiovascular Research Center, Hormozgan University of Medical Sciences, Bandar Abbas, Iran; ^3^ Department of Cardiac Surgery, Behsat Hospital, School of Medical Science, Tehran, Iran

**Keywords:** Coronary Arteries Bypass Graft, Complications, Thrombosis

## Abstract

***Introduction:*** Cardiovascular diseases contribute to mortality and morbidity in aged individuals. It is crucial to have a clear perception of coronary artery bypass graft (CABG) risks and benefits to make logical decision in aged patients. Unfortunately, cardiovascular disease researches have focused very little on the aged patients. The aim of the present study is to evaluate in-hospital complications in patients older than 70 years old following CABG operation to determine if CABG is preferred or not considering present complications.

***Methods:*** In a cross sectional study, 500 patients older than 70 years old were randomly selected (70-75 patients for each year) from March 2004 to March 2011. Descriptive statistical methods were used for evaluating the obtained data.

***Results:*** Overall, 70.6% of patients (353 individuals) were male and 29.4% were female (147 individuals). Totally, 107 patients (21.4%) had complications during hospitalization; these complications were statistically significant in male individuals. Complications included Stroke 1.6%, deep vein thrombosis 0.8%, MI 2.4%, repeat surgery 2.80%, bleeding 2.40%, and more than 48 hours mechanical ventilation in 13.4%.

***Conclusion:*** Need for more than 48 hours mechanical ventilation and bleeding after surgery were the most occurred complications in these patients.

## Introduction

Cardiovascular diseases contribute to mortality and morbidity in aged individuals; aging population in USA leads to increase in the number of the elderly patients with symptomatic coronary artery disease (CAD) most of which would require coronary artery bypass graft surgery (CABG). Consequently, the number of CABG operations is increasing among the elderly patients. It is crucial to have a clear perception of CABG risks and benefits to make logical decision in aged patients. Unfortunately, cardiovascular disease researches have focused very little on the aged patients.^1,2^

Different mortality rates have been reported in studies ranging from 8%-24%; postoperative stroke and renal failure incidence have been reported to be 2%-9% and 2%-13%, respectively.^3^ The definition of aged people in the field of cardiac surgery has increased from 65 to 80 years during the past 20 years.^4^ This is mostly due to the decline in operation risks thanks to the advances made in the technology, methods and proper selection of patients. It is estimated that 6.2 million heart operations are performed in the United States every year 516000 of which are CABG.

The mortality rate of the cardiac diseases in Iran is high and is the first cause of mortality before accidents and cancer.^5^ CAD is defined as the stenosis in all or parts of the coronary arteries due to atherosclerosis or presence of clot; consequently, oxygen cannot be delivered to the myocardium and angina pectoris or infarction occur.^6^ CABG is an effective approach for eliminating or reducing angina pectoris symptoms.^7,8^ Stroke and cognitive disorders following CABG are related to brain embolism.^9^ Acute renal failure occurs in more than 40% of patients 1% of which would require dialysis.^10^

CABG is a surgical treatment that blocked coronary arteries and arteries with stenosis are restored by venous grafts from saphenous vein. The surgical treatment is one of treatment improvements ways which reduce death rates. CABG may be associated with several complications. Aging may not be considered as an independent contraindication in patients undergoing CABG.^10^ Manipulation of ascending aorta is one of the most important factors associated with embolism. Partial clamping of ascending aorta for anastomosing saphenous vein on aorta leads to create 28% of embolisms during operation. Acute renal failure occurs on more than 40% of patients and leads to dialysis in 1% of patients. Development of acute renal failure is associated with a rise in mortality risk, hospitalization period, need for further medications and increase in total cost. In post CABG patient several pathophysiological mechanisms are involved in the development of acute renal failure.^10^

## Materials and Methods

The aim of the study was to evaluate in-hospital complications in CABG patients older than 70 years to determine the risk benefit ratio of CABG in this age group. In a cross-sectional study, 500 patients older than 70 years old who had undergone CABG in Shahid Madani hospital from March 2004 to March 2011 were randomly included (70-75 patient from each year). Complications such as hospitalization time, stroke, and renal failure were evaluated.

Data were analyzed using SPSS version 16. Descriptive statistical methods were used for evaluating data. Chi-square and Fisher exact test were used for qualitative data. *P* value less than.05 was considered statistically significant. Data were extracted from subjects’ hospital files. Private information of patients was kept confidential.

## Results

Mean of patients’ age was 74.86 ± 3.12 years; 70.6% of patients (353 individual) were male and 29.4% female (147 individual). In angiographic studies, 26 patients (5.2%) had one involved vessel; 114 patients (22.8%) had 2 involved vessels and 359 patients (71.8%) had 3 involved vessels. Mean duration of hospitalization after CABG was 9.59±0.33 days with maximum and minimum of 75 and 1 day, respectively. Mean duration of hospitalization in ICU was 3.4 ± 1.12 days with maximum and minimum time of 5 and 1 day, respectively (Table 1).

**Table 1:** Angiography Results of the Study Patients

**Vessel Involvement**	**No. (%)**
1 involved vessel	26 (5.2)
2 involved vessel	144 (22.8)
3 involved vessel	359 (71.8)
More than three involved vessel	1 (0.2)

**Figure 1:**
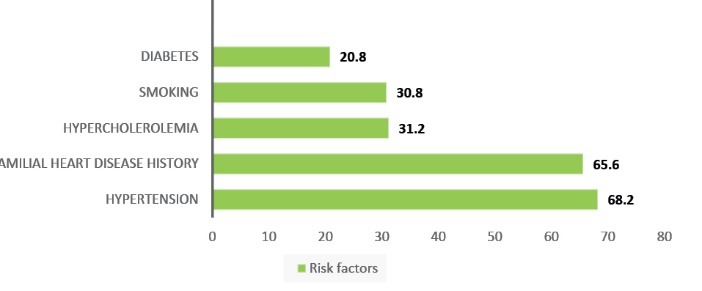
The Most Studied Risk Factors.

The most studied risk factors was illustrated on Figure 1. Totally, 107 patients (21.4%) had several complications during hospitalization; these complications were statistically significant in male individuals (*P *= .022) and include MI (9 female, 19 male, 5.6%).

**Figure 2:**
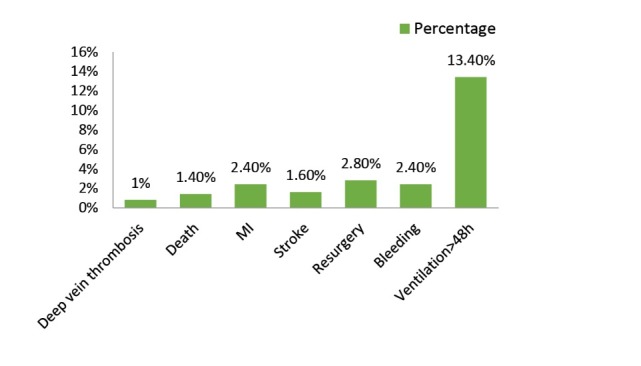
The Rate of Complications After CABG Operation During Hospitalization.

Deep vein thrombosis was observed in four patients (0.8%) while 7 cases (1.4%) of intra-hospital death were reported. Accordingly, 28 patients‏ (5.6%) had myocardial infarction (MI) (9 female and 19 male subjects); 16 patients before operation and 12 patients after operation had MI; 8 patients (1.6%) had stroke during hospitalization. 14 patients (2.8%) needed second operations, 12 patients (2.4%) had bleeding during their hospital stay and 13.4% of patients (67 patients) required more than 48 hours of ventilation or reintubation (Figure 2). Bleeding in men was more frequent than women during hospitalization (*P *= .02). The rate of CABG complications was higher in individuals older than 70 years and with hypercholesteremia (*P *= .024). Also, complications had a significant correlation with the familial history of heart disease (*P *= .013). The rate of CABG complications was significantly higher in subjects with history of MI (*P *= .0001), respiratory disease (*P *= .000), renal disease (*P *= .000), stroke (*P *= .02) and diabetes (*P *= .009). There was significant relation between diabetes and death in hospital (*P *= .001).

Ejection fraction (EF) on 310 patients (62%) was more than 40%; 150 patients (30%) has between 30%-40% EF and 40 patients (8%) has less than 30% EF. Among patients, 107 individuals (21.4) has different symptoms during hospitalization. This complications in males significantly were more than females (*P *= .022). Deep vein thrombosis is seen in 4 patients (0.8%) and death entire hospital in 7 patients (1.4%).

There was significant relation between diabetics and need for ventilation more than 48 hours (*P *= .009). The rate of complications in diabetics was significantly higher than others (*P *= .001). Also, there was significant relation between hypercholesteremia and second operation (*P *= .034), bleeding (*P *= .007) and ventilation more for more than 48 hours (*P *= .044).

In our study, there was no significant relation between stroke and diabetes (*P *= .241) or hypertension (*P *= .677).

## Discussion

Cardiovascular diseases are very common and highly contribute to the death of aged people. Several factors are involved in the occurrence of atherosclerosis such as obesity, hypertension, smoking, hyperlipidemia, and diabetes. Coronary artery bypass surgery is effective method for treatment and removing of pectoral angina; consequently, it declines death rate.

Previous studies reported low rate of success with high complications.^11-13^ During a clinical trial, surgical treatment was compared with medical treatment; CABG subjects older than 75 years had less complications and better quality of life.^14^ Mortality after CABG in Babatabar Darzi et al^13^ was reported to be 1.5%. In our study, the rate of mortality after CABG was 1.4%. Boeken et al^14^ reported the rate of neurologic complications following CABG to be 1.7%. Alexander et al^15^ study reported a 1.4% stroke incidence after operation. Stroke following CABG in our study was reported in 1.6% of the cases. Stamou et al^16^ reported a significant relation between post-CABG stroke and hypertension, age more than 75 years and diabetes. In the present study, post-CABG hemorrhage was reported in 2.4% of the subjects. While, Babatabar Darzi et al^13^ reported 6% of hemorrhage after CABG.

Alexander et al^15^ reported an incidence of 2.9% for acute renal failure in their studied subjects. This point in Babatabar Darzi et al^13^ study was 16.5%. The figures for our study were 6.4%.

Results showed that 13.4% of patients required ventilation after 48 hours. Babatabar Darzi et al^13^ reported 8.7% of pulmonary complications. Consequently, Horneffer et al^17^ presented that individuals with more than 70 years need more ventilation after CABG.

## Conclusion

CABG like other invasive treatment methods has its own symptoms. Our results demonstrated that the need for more than 48 hours ventilation was the most occurred complication while deep vein thrombosis was the least occurred. Therefore, taking care before operation and precise monitoring of subjects for declining complications is necessary.

## Ethical Issues

The study was approved by the ethical committee of Tabriz University of Medical Sciences.

## Conflict of Interests

The authors declare no conflict of interests.
